# A case of double cystic esophageal duplication in VACTERL syndrome: the first case report and a review of the literature

**DOI:** 10.3389/fped.2023.1151039

**Published:** 2023-04-19

**Authors:** G. Vasta, S. Tursini, E. Rovero, R. Angotti, F. Molinaro, V. Briganti

**Affiliations:** ^1^ Pediatric Surgery, San Camillo-Forlanini Hospital, Rome, Italy; ^2^ Pediatric Surgery, Department of Medical, Surgical and Neurological Sciences, S. Maria Alle Scotte Hospital, University of Siena, Siena, Italy; ^3^ Azienda Ospedaliera Universitaria Meyer, Firenze, Italy

**Keywords:** esophageal duplication cyst, VACTERL, case report, thoracoscopy, pediatric surgery

## Abstract

**Background:**

An esophageal duplication cyst (EDC) is a rare malformation resulting from the embryonic foregut. VACTERL syndrome is a genetic disorder affecting many systems of the human body. We report the first case of VACTERL syndrome associated to asymptomatic double EDC.

**Case report:**

A girl with anorectal malformation and rectovestibular fistula, kidney malformation, and various vertebral defects came to our attention at the time of birth. VACTERL disease was diagnosed. She underwent Peña anoplasty at 4 months of life without complications. MRI was conducted at the age of 2. It accidentally showed a double esophageal duplication (12 mm × 35 mm × 10 mm) at the D7–D9 level. We planned a thoracoscopy; previous intraoperative esophagogastroduodenoscopy showed an external compression of the native esophagus. Two duplicated esophageal lesions were removed. The patient made an uneventful recovery and was completely asymptomatic at long-term follow-up.

**Conclusions:**

VACTERL syndrome is still a not well-defined disease. Based on the current literature, this is the first case of a double esophageal duplication in a patient affected by VACTERL syndrome. According to us, the thoracoscopic approach of esophageal duplications can be followed by experts. Complete surgical excision is possible even if the cyst shares a common muscular wall with the esophagus. For this reason, we suggest to close the muscular wall by a simple interrupted suture.

## Introduction

The EDCs (congenital esophageal duplication cysts) are rare congenital anomalies associated to other congenital defects such as esophageal atresia, intestinal duplication, and tracheoesophageal fistulas (1). The incidence of EDCs is estimated at 1:8,200, with 2:1 male predominance (2, 3).

Foregut cysts can be classified as follows: bronchogenic, heterogenous (which include esophageal duplication), neurenteric, or mixed (4). EDCs are surrounded by smooth muscle on a double layer and lined by alimentary epithelium (squamous or enteric). They either attached to the esophagus in a paraesophageal or intramural way (2, 5–7).

We herein report a unique case of an asymptomatic double cystic esophageal duplication associated with VACTERL syndrome without esophageal atresia.

Fabris et al. described a similar case of a patient affected by VATER syndrome with unique esophageal cystic duplication that differs from our case for the esophageal atresia (8).

## Case description

**Figure 1:**
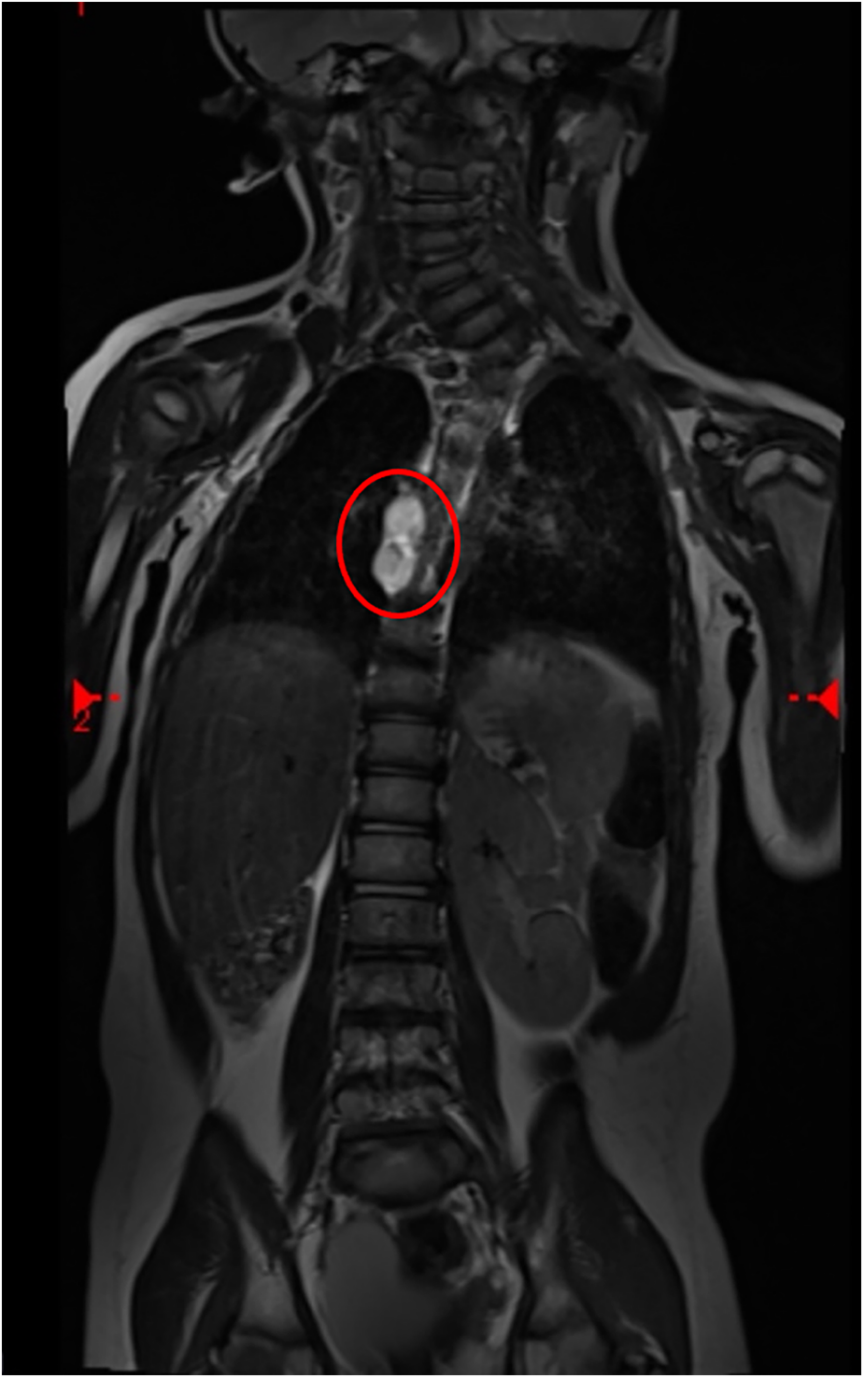
MRI scan of cystic lesions of the esophagus (circle: EDC at the D7–D9 level).

A girl with anorectal malformation (rectovestibular fistula), kidney malformation, and various vertebral defects came to our attention at birth without prenatal screening. VACTERL syndrome was diagnosed. She underwent Peña anoplasty at 4 months without any complications. MRI was conducted at the age of 2 to follow up vertebral defects. MRI accidentally showed a double esophageal duplication (12 mm × 35 mm × 10 mm) at the D7–D9 level (Figure 1). We decided to perform surgery preceded by an esophagoscopy that showed an external compression of the native esophagus under aortic narrowing.

The patient was placed in the lateral decubitus position with the involved side up. A round bolster was placed under the thorax to arch the vertebral column upward to maximize the intercostal spaces of the involved side.

Three trocars were placed: a 5-mm trocar for the optic was put on the fifth intercostal space in the medium axillary, line while two 3-mm operative trocars were positioned on the same intercostal line on the anterior and posterior axillary lines, respectively.

The thorax was insufflated with CO_2_ with a pressure of 3 mmHg. We did not use single lung ventilation or any ancillary trocar to retract the lung.

**Figure 2:**
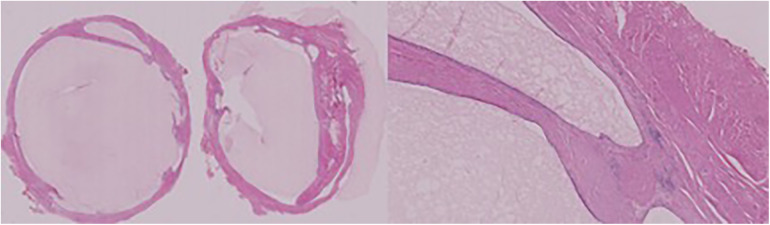
Left: EDCs; right: details of each layer.

We totally removed the double cists that were on the third distal part of the esophagus, with minimally invasive procedure, by using monopolar and blunt dissection to preserve the vagus nerve, the azygos vein, and the esophageal wall. We closed the muscular defects after excision using separate and absorbable stitches (3-0 Vicryl), and we left thorax drainage (Figure 2).

**Figure 3:**
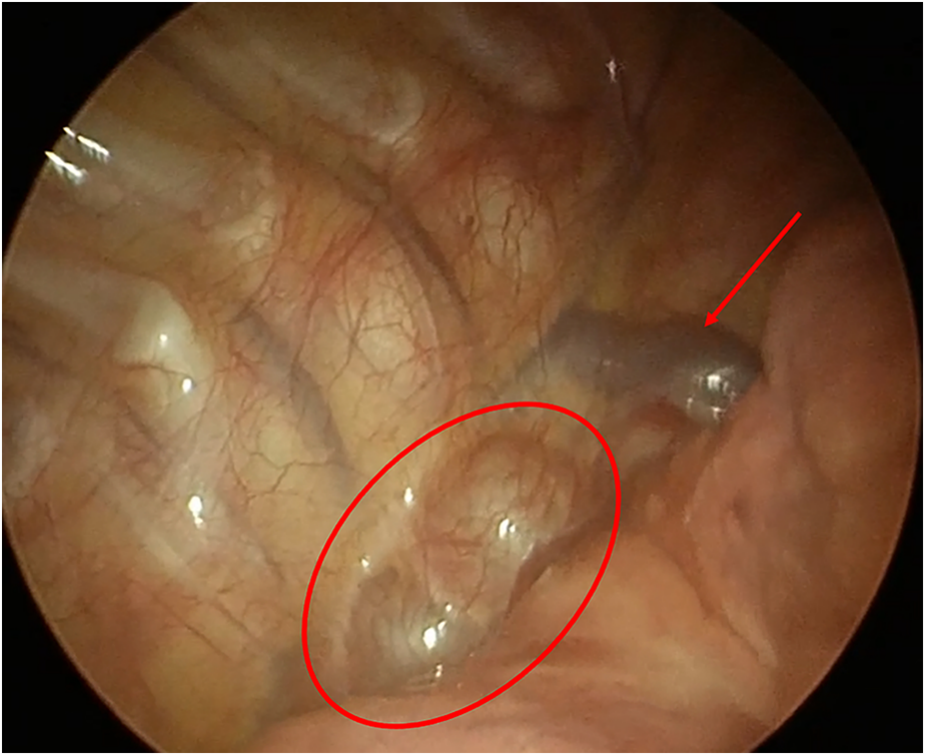
Foregut duplication cysts. Intrathoracic picture (arrow: azygos vein; circle: EDCs).

The postoperative evolution was uneventful. We removed the drainage, and the patient started oral feeding after 24 h from the surgery. She was discharged after 4 days without any complications. The histological report confirms the diagnosis of double cystic esophageal duplication (Figure 3).

**Figure 4:**
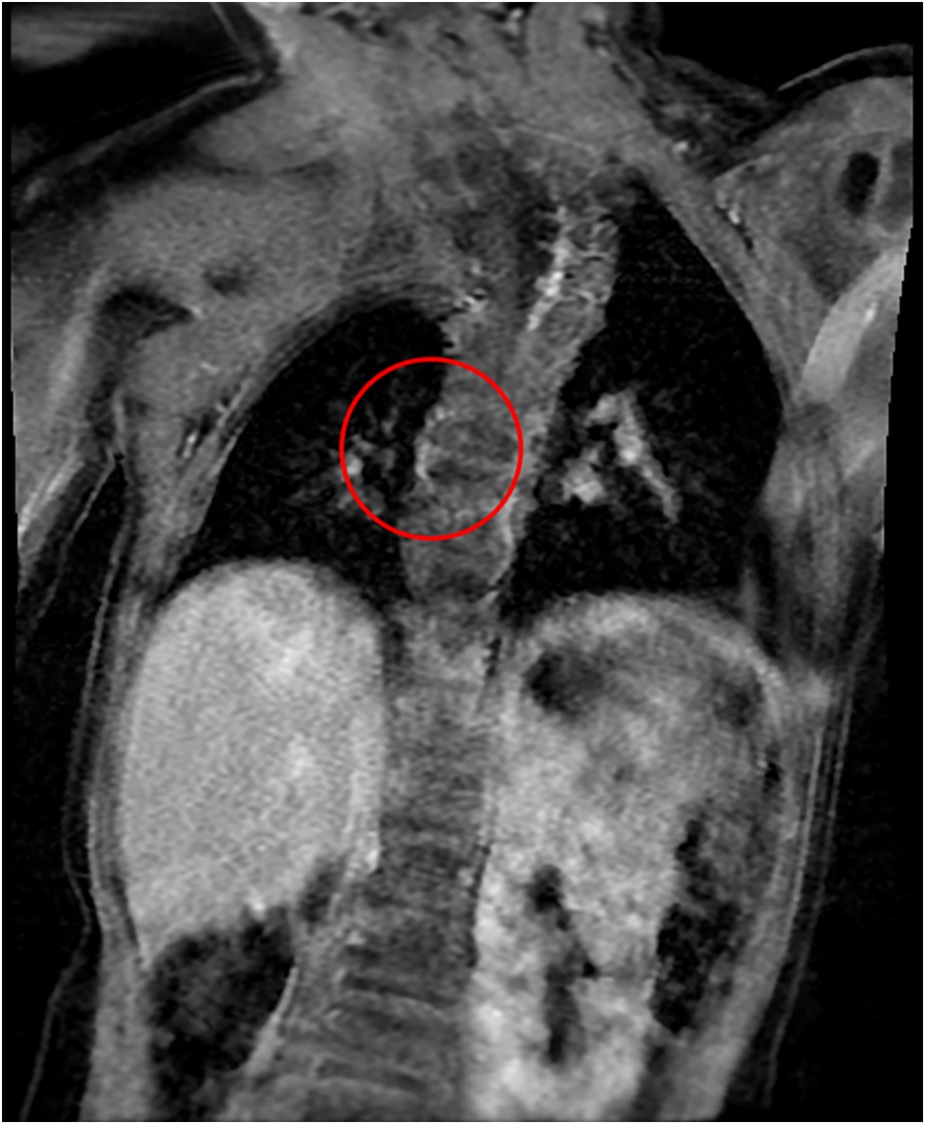
MRI scan 16 months after surgery (the circle shows no lesions).

After 12 months of follow-up, the patient showed any GI symptoms, and oral feeding was compliant Sixteen-month MRI showed normal anatomy of the esophagus (Figure 4).

## Discussion

Among the mediastinal cysts, EDCs are the most common They might be result of abnormal development of the primitive foregut. They are epithelial-lined structures defined by histologic features and not by their position. Foregut cysts can be classified as follows: bronchogenic, heterogenous (which includes esophageal duplication), neurenteric, or mixed (9).

Most of the esophageal duplication cysts are located in the right posterior-inferior mediastinum. Two-thirds of these lesions are found in the lower third of the esophagus and the other third in the upper/middle third of the esophagus (10).

CT and MRI scans are tools used to study mediastinal masses; however, several types of imaging share similar features.

The clinical presentation of EDCs depends on the dimension and infection of the cyst. About 80% of these lesions are diagnosed in childhood, and the majority are symptomatic such as pulmonary infection, respiratory distress, dysphagia, and nutritional intolerance (5, 11). Most of these cysts are benign, with asymptomatic anomalies that occur during foregut formation; neurological complications are usually the reasons for initial investigation (12).

Early surgical resection is recommended for both symptomatic and asymptomatic patients with EDCs. Developmental aberrations can result from recurrent or persistent pulmonary infections. It is recommended to perform an early elective surgery since the increased rate of infection might make the surgery more difficult (13).

The thoracotomy approach has been the standard of care for the excision of EDCs. However, in recent years, it has been proven that the thoracoscopic approach has been described to be safer for this indication (14–16), since it offers advantages such as reduced postoperative pain, shortened length of hospital stay, and improved cosmesis. In addition, the morbidity of a thoracotomy can be avoided (17).

The literature describes tissue dissecting devices using ultrasound or thermal energy for removal through enucleation or partial resection (14, 17–19).

## Conclusion

VACTERL syndrome is still an unknown disease since the specific genetic mutation is unknown. The literature describes a few cases of esophageal duplication related to esophagus atresia in patients without VACTERL syndrome (20–24).

Only one case is described by Fabris et al. in 1995 similar to our patient, affected by VACTERL syndrome and esophageal foregut duplication. The patient described had esophageal atresia with a unique EDC (8). Therefore, our case is the first that describe a double cystic esophageal duplication in a VACTERL syndrome without esophageal atresia.

Thoracoscopic removal of ECD is safe, with enormous advantages over thoracotomy.

## Data Availability

The raw data supporting the conclusions of this article will be made available by the authors, without undue reservation.
